# T-cell immunity to SARS-CoV-2: what if the known best is not the optimal course for the long run? Adapting to evolving targets

**DOI:** 10.3389/fimmu.2023.1133225

**Published:** 2023-06-14

**Authors:** Alexandre E. Nowill, Manuel Caruso, Pedro O. de Campos-Lima

**Affiliations:** ^1^ Integrated Center for Pediatric OncoHaematological Research, State University of Campinas, Campinas, SP, Brazil; ^2^ CHU de Québec-Université Laval Research Center (Oncology Division), Université Laval Cancer Research Center, Québec, QC, Canada; ^3^ Boldrini Children’s Center, Campinas, SP, Brazil; ^4^ Molecular and Morphofunctional Biology Graduate Program, Institute of Biology, State University of Campinas, Campinas, SP, Brazil

**Keywords:** COVID-19, SARS-CoV-2, vaccine, T cells, immune resetting

## Abstract

Humanity did surprisingly well so far, considering how unprepared it was to respond to the coronavirus disease 2019 (COVID-19) threat. By blending old and ingenious new technology in the context of the accumulated knowledge on other human coronaviruses, several vaccine candidates were produced and tested in clinical trials in record time. Today, five vaccines account for the bulk of the more than 13 billion doses administered worldwide. The ability to elicit biding and neutralizing antibodies most often against the spike protein is a major component of the protection conferred by immunization but alone it is not enough to limit virus transmission. Thus, the surge in numbers of infected individuals by newer variants of concern (VOCs) was not accompanied by a proportional increase in severe disease and death rate. This is likely due to antiviral T-cell responses, whose evasion is more difficult to achieve. The present review helps navigating the very large literature on T cell immunity induced by severe acute respiratory syndrome coronavirus 2 (SARS-CoV-2) infection and vaccination. We examine the successes and shortcomings of the vaccinal protection in the light of the emergence of VOCs with breakthrough potential. SARS-CoV-2 and human beings will likely coexist for a long while: it will be necessary to update existing vaccines to improve T-cell responses and attain better protection against COVID-19.

## 1 Introduction

When the first cases of pneumonia were identified in China at the end of 2019, little was known about the new illness, which was later named coronavirus disease 2019 (COVID-19) (1). It was soon found that COVID-19 was caused by an enveloped positive-sense, single-stranded RNA virus that belongs to the *Betacoronavirus* genus – the severe acute respiratory syndrome coronavirus 2 (SARS-CoV-2) (2). An unprecedented scientific effort led to the development and testing of several vaccine candidates, which culminated with their regulatory approval and the administration of billions of doses worldwide (**Table 1**).

**Table 1:** Protection from symptomatic disease by vaccines with the largest population coverage at the time of first authorized use.

Vaccine	Manufacturer	Dosing	First emergency use authorization/ listing	Clinical trial considered for first use authorization	Participants included in the first efficacy analysis ^a^	Efficacy at the time of first authorized use	Deployment (Doses in billions)
**BNT162b2**	Pfizer-BioNTech	2 doses	MHRA, UK Dec 02, 2020	NCT04368728	36,523	95.0%	> 2.5
**mRNA-1273**	Moderna	2 doses	FDA, USA Dec 18, 2020	NCT04470427	28,207	94.1%	> 0.5
**AZD1222**	Oxford/Astra-Zeneca **^b^**	2 doses	MHRA, UK Dec 30, 2020	ISRCTN89951424, NCT04324606, NCT04400838, NCT04444674	11,636	70.4% **^c^**	> 3.0
**CoronaVac**	Sinovac	2 doses	SAGE-WHO Jun 01, 2021	NCT04456595, NCT04582344, NCT04508075	9,823 10,029 1,620	50.6-83.5% **^d^**	> 2.9
**BBIBP-CorV**	Sinopharm	2 doses	SAGE-WHO May 07, 2021	NCT04510207	27,530	78.1%	> 2.5

**a:** Per Protocol Population.

**b:** The same formulation is also produced by the Serum Institute of India as Covishield and was approved by a separate regulatory path.

**c:** Median value used by the Agency from ISRCTN89951424 and NCT04400838 clinical trials (3, 4). Later, it was reported that two doses spaced by a 3-month-long dosing interval had an efficacy of 81.3% (5).

**d:** CoronaVac efficacies were: 50.6% for NCT04456595 (6), 65.3% for NCT04508075 (7) and 83.5% for NCT04582344 (8). The table was compiled from references (9) (NCT04368728) (10), (NCT04470427) (3), (ISRCTN89951424, NCT04400838) (6), (NCT04456595) (7), (NCT04508075) (8), (NCT04582344), and (11) (NCT04510207), as well as (4, 12–14). MHRA: Medicines & Healthcare products Regulatory Agency; FDA: Food & Drug Administration; SAGE-WHO: Strategic Advisory Group of Experts, World Health Organization.

From the population standpoint, the immune status changed considerably in a short time, evolving from a situation in which most human beings were immunologically naïve to SARS-CoV-2 to another in which some remained naïve and some were primed by infection. A third group soon emerged who had post-vaccinal immunity. Other layers of complexity were then added by transitional cases who had priming by infection followed by “boosting” by vaccination – known as hybrid immunity (15). The latter comes in several flavors that reflect: **(i)** the viral strain variant that caused the primary infection (16); **(ii)** the type of vaccine (there are 5 major platforms that will be described later); and **(iii)** the individual immunization history (complete or incomplete vaccination cycle with or without booster shots). Other relevant scenarios were created by reinfections or breakthrough infections in vaccinated individuals by immune evasive variants, such as omicron (16, 17). Finally, cross-reactivity to other coronaviruses may also impact on the SARS-CoV-2-specific immune response (18).

There is strong rationale for the adoption of COVID-19 immunization strategies that aim primarily at developing robust antibody responses with an effective neutralizing component in order to limit viral spread in the community. Much attention has justifiably been given to this topic in the literature (19–21). However, mounting evidence points to a major role of T cells in the protection conferred by infection and vaccination. Hence, SARS-CoV-2-infected mice expressing human angiotensin-converting enzyme 2 (ACE2) induced strong polyfunctional CD4^+^ and CD8^+^ T cell responses that could dump viral titers in the lungs (22). Indeed, vaccination in this model could protect the animals from infection in the absence of neutralizing antibodies. Experiments conducted with convalescent rhesus macaques revealed that depletion of T cells with anti-CD8 antibodies led to breakthrough infection upon SARS-CoV-2 rechallenge (23). As regards clinical data, earlier studies reported a broader and stronger T cell response in COVID-19 patients with severe disease (24, 25). It remains unknown if this possibly uncoordinated and dysfunctional reaction just reflects higher viral loads in the advanced stages of the disease and/or is part of its pathogenesis (sepsis). Moreover, one of these initial reports identified a protective role of the CD8^+^ subset alongside a lower total T cell response in mild disease (24). Nevertheless, it is worth noting that the results of many other studies are in line with the inferred protection indicated by the mentioned pre-clinical findings. Thus, it was shown that early virus-specific T-cell induction improves viral clearance and COVID-19 prognosis (26). A recent report assigned this protective effect to CD4^+^ T cells which was mostly evident in the first 2 weeks of infection (27). Another study described the participation of both T cell subsets in limiting disease severity but found that IFN-γ-producing CD8^+^ T lymphocytes exhibit the strongest association with milder acute COVID-19 (28). In agreement with these findings, CD8^+^ T cells limit viral load, disease severity, and mortality in COVID-19 patients with hematologic malignancies undergoing B cell depletion by anti-CD20 therapy (29). Also, clonally expanded CD8^+^ T lymphocytes were described in the bronchoalveolar lavage fluid in COVID-19 moderate cases (30).

Altogether, the emerging picture reveals the importance of cellular immunity in COVID-19 – notably in those circumstances in which variants of concern (VOCs) may breach the barrier created by antiviral neutralizing antibodies. This article intends to revisit the infection- and vaccine-induced SARS-CoV-2-specific adaptive cellular immunity with a focus on T lymphocytes. For in-depth analysis of antiviral cellular or humoral immunity including prophylactic and therapeutic implications, the reader is directed to several excellent reviews published elsewhere (19–21, 31–37).

## 2 Preparing the ground for the adaptive immune response to SARS-CoV-2 primary infection

Pattern recognition receptors (PRRs) sense pathogen-associated molecular patterns (PAMPs) thereby activating the first line of defense against viral infection in naïve individuals (38, 39). Thus, the toll-like receptors (TLRs) 7 and 8 are capable of identifying uridine-rich, single-stranded RNA within the endosomal compartment, thereby triggering through the myeloid differentiation primary response 88 (MyD88) adaptor the transcription of genes encoding type-I interferons (IFNs) as well as major proinflammatory cytokines, such as IL-1β and IL-18 (38, 40). The latter cytokines are produced as larger nonfunctional precursor molecules that are recognized and activated by the NLR family pyrin domain containing 3 (NLRP3) inflammasome (41). Similarly, the cytosolic sensors retinoic acid-inducible gene I (RIG-I), melanoma differentiation-associated protein 5 (MDA5), laboratory of genetics and physiology 2 (LGP2), and the nucleotide-binding oligomerization domain-containing protein 1 (NOD1) were all shown to engage SARS-CoV-2 RNA resulting in the production of type-I IFNs (39). Most cells may be a source of these cytokines, including those lining the respiratory tract, albeit not to the level achieved by plasmacytoid dendritic cells (DCs) (42).

Regardless of their origin, type-I IFNs engage their ubiquitous receptor (IFNAR) and initiate the signal transducer and activator of transcription (STAT) 1/2-mediated transcription of a plethora of interferon-stimulated genes (ISGs) – many of them encoding products that act directly or indirectly to contain viral infections (42). There are multiple examples of ISG products that counteract SARS-CoV-2 during distinct segments of the viral cycle: **(i)** in the entry phase (e.g., E74 like ETS transcription factor 1); **(ii)** in the translation/replication phase (e.g., Z-DNA-binding protein 1 and IFN-induced protein with tetratricopeptide repeat 3), and **(iii)** in the release phase (e.g., tetherin) (43).

In addition to their direct antiviral effect, type-I IFNs also enhance the ability of individual cells to recognize and respond to pathogens early on during infection. This is achieved by the upregulation of a subset of ISG products that act operationally as PRR receptors (42). Among those ISGs relevant to SARS-CoV-2, it is possible to include the ones which encode RIG-I, MDA-5, oligoadenylate synthetase-latent endoribonuclease L, and TLR-7 (42, 44). It is conceivable that higher expression of these PRRs would sound faster the alarm against infection, perhaps requiring lower viral loads, and allowing the implementation of the IFN effects with direct antiviral impact.

Given the importance of type-I IFNs in shaping the early phase of viral infection, it results unsurprising that more than a third of the SARS-CoV-2 genome should be dedicated to encode over a dozen products, including several nonstructural (NSPs) and accessory (ORFs) proteins, which may directly or indirectly disable IFN production or its receptor activity (39).

Overall, the activation of PRRs serve multiple purposes: (i) to contain the virus in the infected cell; (ii) to inform other cells to do the same; (iii) to promote the release of proinflammatory cues (e.g., IL-1β, IL-18, IL-6, IL-12, TNF-α, and IFN-γ) notably by immune cells which activate further the innate response locally and systemically; and (iv) to prepare the ground for the generation of a targeted, and often resolutive, adaptive response through the activation of professional antigen presenting cells (APCs) (38, 39, 42, 45).

Depending on the viral load during the initial contact as well as the pathogen genetics, the infection may be aborted early on by an innate immune reaction or by a pre-existing cross-reactive adaptive memory response (33, 46–48). Nevertheless, should the infection surpass this stage and get established, T cells are required for viral clearance (49). Conventional T cells derive from bone marrow progenitors which migrate to the thymus where they undergo T cell receptor (TCR) recombination, followed by positive and negative selection (50). Once graduated from thymic maturation, MHC class I-restricted CD8^+^ and MHC class II-restricted CD4^+^ cells exit from the lymphoid organ into the circulation as quiescent CD45RA^+^CCR7^+^ naïve T lymphocytes (50, 51). Their highly diverse TCR repertoire comprises close to 10^8^ specificities warranting potential recognition of virtually all SARS-CoV-2-encoded T-cell epitopes in the context of a given HLA haplotype – provided that no relevant repertoire deletion had been imposed on the epitope specificities in question (51). The fact that the thymus operationally ends its function in the adult after the fourth decade of life is compensated by the 5-10-year-long lifespan of the naïve cells and by their renewability in the periphery (52). Yet, there is evidence that the scarcity of naïve T cells, particularly naïve CD8^+^ T lymphocytes, favors COVID-19 severity in the elderly (28). In any case, regardless of being originated from the thymus or from peripheral turnover, naïve cells undergo a three-phase differentiation process upon activation that comprises: **(i)** clonal expansion, **(ii)** contraction, and **(iii)** memory formation.

Clonal expansion is mediated by IL-2 that triggers an autocrine cell cycle activation (52). It is complemented by further differentiation that provides T cells with effector tools to control viral infections. The prototypic immune response that is associated with an eventual virus clearance is mediated by CD8^+^ cytotoxic T lymphocytes (CTLs) (49). The latter recognize antigenic peptides derived from intracellular pathogens presented by MHC molecules on the surface of the infected cell, unleashing the release of lytic granules and effector cytokines, such as IFN-γ and TNF-α (53). The CD4^+^ effector counterparts act in unison assuming several polarization helper phenotypes associated with classical cytokine secretion patterns: **(i)** IFN-γ-producing Th1 cells are pleiotropic, and favor the antiviral action of CTLs (54); **(ii)** Th2 cells produce IL-4, IL-5 and IL-13, acting on some granulocytes and B cells (50); **(iii)** Th17 cells are proinflammatory and produce IL-17A/F and IL-21 (55); **(iv)** Two minor subsets – Th9 (IL-9) and Th22 (IL-22) – exhibit some overlap with Th2 and Th17 cells, respectively (50); **(v)** T follicular helper (T_FH_) cells provide essential support to coordinate B cell proliferation, survival, and differentiation into antibody-producing plasma cells (56). Thus, co-signaling through the axis inducible T-cell co-stimulator (ICOS) ligand-ICOS enhances T-B cell entangled contacts in the germinal centers and upregulate the CD40L in T_FH_ cells, which together with the production of IL-21, ultimately favor affinity maturation in memory B cells (50, 56); and **(vi)** Regulatory T cells (T_REG_s) are primarily thymus-derived but may differentiate from effector cells in the periphery as CD4^+^ CD25^+^ Foxp3^+^ T lymphocytes (57). In the blood and lymphoid tissues, they maintain a CD45RA^+^ CCR7^+^ naïve phenotype but acquire a CD45RA^-^ CD45RO^+^ memory-like expression profile in mucosal sites. They suppress immunity through direct cell-cell contact and secretion of IL-10 and TGF-β (52, 57). Two further points about the above-described phenotypes are worth consideration. First, analogous CD8^+^ subsets to those described for CD4^+^ T cells (Tc1, Tc2, Tc17, and T_REG_) do exist (58, 59). Second, in addition to the classic cytokine secretion profiling, activation marker expression may be used to identify T cell subsets by flow cytometry as it will be described later in the text for SARS-CoV-2 epitope-specific T cells.

Once infection is resolved, effector T cells experience massive contraction by apoptosis. A small fraction of them survives as CD45RA^+^ CCR7^-^ terminally differentiated effector memory cells re-expressing CD45RA (T_EMRA_). These cells are most often CD8^+^, secrete good amounts of IFN-γ, have limited proliferative capacity, and their relative frequency depends on viral load and pathogen persistence (52). The remainder of the surviving cells acquire one of three memory phenotypes: CD45RA^-^ CCR7^+^ central memory (T_CM_), CD45RA^-^ CCR7^-^ effector memory (T_EM_), and CD45RA^+^ CCR7^+^ CD95^+^ CD122^+^ stem cell memory (T_SCM_) (60, 61). T_CM_ cells are more proliferative, respond to homing cues to lymph nodes, have limited immediate effector function but differentiate into T_EM_ upon secondary stimulation. Conversely, T_EM_ cells can migrate to inflamed tissues and activated lymph nodes, and may exhibit immediate effector function (61, 62). T_SCM_ cells are much less frequent. They do not have immediate effector function but display high proliferative potential and are capable of self-renewal (60).

Attempts to characterize virus-reactive T cell memory subsets have often had the bias of limiting the analysis to circulating cells in the peripheral blood – including most studies on SARS-CoV-2. Nevertheless, an important contingent of memory cells follow chemokine gradients towards epithelial surfaces, lose their ability to respond to tissue exit cues, and become tissue-resident memory (T_RM_) lymphocytes. Most of them express CD69 that downregulates sphingosine 1-phosphate (S1P) receptors, cutting their ability to respond to egress factor cues (63). T_RM_ cells exhibit a dual regulatory and effector profile. On the one hand, T_RM_ cells express PD-1, LAG3, and CTLA-4, and secrete IL-10 which limits overactivation – but on the other hand, they can secrete IFN-γ, TNF-α, IL-17 and IL-2 unleashing a quick *in situ* response to invading pathogens (52).

Many of the above-described T cell subsets will be revisited in the next sections in a SARS-CoV-2 context.

## 3 T-cell immunity in SARS-CoV-2 primary infection

By using orthogonal approaches, including activation-induced marker (AIM) analysis, intracellular cytokine staining, ELISPOT, and tetramer staining, it is possible to detect CD8^+^ T lymphocytes in the peripheral blood of about 70% of COVID-19 convalescent patients one month after infection. However, this detection drops to about 50% six months later (64). The kinetic profile shows expansion in the first month and a gradual decrease thereafter with an estimated half-life (t_1/2_) within the window of 125-196 days (64, 65). Although all viral proteins are potential targets in the right MHC context, these CD8^+^ T lymphocytes recognize well in the AIM assay at least eight viral antigens (NSP3, NSP4, NSP6, NSP12, S, ORF3a, membrane glycoprotein, and nucleoprotein) and tend to focus on four dominant targets – three of them highly expressed (spike, membrane glycoprotein, and nucleoprotein) and one weakly expressed (NSP3) (64, 66). The intracellular cytokine assay reveals an even narrower targeting as close to 60% of SARS-CoV-2 CD8^+^ T-cell responders recognize the nucleoprotein, and 43% of the total CD8^+^ T cell-response in each individual is dominated by this specificity (65). On average, each donor reacts to at least 17 CTL epitopes (66).

During acute infection, SARS-CoV-2-reactive CD8^+^ T cells have a highly cytotoxic phenotype with perforin and granzyme B, as well as show clear signs of activation and proliferation given by the expression of CD38, CD69, HLA-DR and Ki-67 (31, 67, 68). Furthermore, the immune checkpoint molecules PD-1, LAG3, TIM-3, CTLA4 and CD39 are all upregulated early on; albeit the expression levels of PD-1 and CD38 drop in late convalescence (67, 68). The latter molecules are usually associated to exhaustion. However, by running intracellular cytokine staining in peptide-stimulated, SARS-CoV-2-specific, MHC class I multimer^+^ CD8^+^ T cells, Rha et al. have shown that the frequency of IFN-γ-producing cells is not different in early and late convalescent samples (68). They also found that the cells retain proliferative capacity upon antigen re-challenge. The findings led these authors to conclude that the phenotype is not one of exhaustion but rather one of activation with preserved effector function. Indeed, current evidence seems to support this interpretation, including a single-cell transcriptomic study that revealed the shrinkage of the “exhaustion” cluster over time, which correlated with cell cycling (69). In addition, SARS-CoV-2-reactive CD8^+^ T cells are often capable of simultaneous production of IFN-γ, TNF-α and granzyme B not only during the early expansion phase but retain this polyfunctionality throughout the following 6 months or more (65).

CD8^+^ T cells have CD45RA^-^ CCR7^-^ effector memory (T_EM_) surface markers during the early phase of the response that are progressively lost. Conversely, a CD45RA^+^ CCR7^-^ CD8^+^ (T_EMRA_) population gradually ascends, while the CD45RA^-^ CCR7^+^ CD8^+^ central memory (T_CM_) cells remain stably low until day 240 post-infection (64, 65, 68). It is worth noting that the expansion of TCF1^+^ T_EMRA_ occurs alongside a detectable CD95^+^ CD45RA^+^ CCR7^+^ CD8^+^ stem cell-like memory (T_SCM_) subset which gradually expands until day 120 of convalescence and stabilizes thereafter (65, 70, 71). Stem cell-like memory is associated with self-renewal capacity and multipotency to repopulate the other memory and effector T-cell subsets (72). Tissue-resident memory T cells (T_RM_) are another often neglected memory subset which may play a major role in local immune protection at mucosal, skin, and various organ sites (52, 73). Poon et al. analyzed samples obtained from four organ donors who died from noninfectious causes but had previously recovered from COVID-19, one of them >6 months earlier (74). These investigators used the CD69 and CD103 markers to identify SARS-CoV-2-specific CD8^+^ T_RM_ cells in the spleen, lung/gut lymph nodes, and lungs, with the highest frequency found in the latter.

About 89%-93% of the people who are clinically infected with SARS-CoV-2 mount a CD4^+^ T-cell memory response against at least one viral structural protein during the first month after disease onset, which declines thereafter but remains detectable for 6-8 months in 92% of the convalescent patients (64, 65). The preferential antigenic targets are: spike, membrane glycoprotein, nucleoprotein, ORF3a, and NSP3 (64). On average, each donor reacts to at least 19 T-cell epitopes (66). Although the number of reactive cells reaches >1% in 42% of the patients at 1 month, the median frequency of circulating virus-specific CD4^+^ T lymphocytes is 0.51% and their estimated t_1/2_ ranges from 94-207 days (64, 65). Nevertheless, the resilience of these memory cells may be even longer. Indeed, Wragg et al. have used peptide-MHC tetramers to track T lymphocytes that recognize the immunodominant SARS-CoV-2 spike epitope (S_751-767_) presented by HLA-DRB1*15:01, and determined their t_1/2_ to be approximately 377 days (75). By the end of this period, the frequency of epitope-reactive cells drops to 0.0038%, which is 3.6 times lower than in the first weeks post-infection but it remains higher as compared to uninfected controls (75).

Upon recognition of SARS-CoV-2-derived cognate epitopes, CD4^+^ T lymphocytes express activation markers (CD38, CD69, Ki-67 and HLA-DR) and turn on a phenotypic program that includes the simultaneous expression of two or more cytokines (often IFN-γ, IL-2 or TNF-α) (65, 67). The memory response was evaluated with the AIM assay which identified among the CD69^+^ CD137^+^ activated subset a clear polarization skewing towards Th1 (CCR4^-^ CCR6^-^ CXCR3^+^ CXCR5^-^) in membrane glycoprotein- and nucleoprotein-specific, as well as to cT_FH_ (CXCR5^+^) in spike-specific CD4^+^ T cells (67). The cT_FH_ phenotype is critical for the generation of binding and neutralizing antibodies. The CXCR5 chemokine receptor allows cT_FH_ cells to migrate to lymph nodes where they contribute to the generation and maintenance of germinal centers through the concerted release of IL-21 and the expression of the CD40L (76). During COVID-19 early convalescence about 10% of the virus-reactive CD4^+^ T cells may also express CXCR5. In fact, HLA-DRB1*15:01-restricted, S_751-767_-specific cT_FH_ lymphocytes were still detectable in 13 out of 17 convalescent patients 365-450 days after symptoms onset (75). The predicted t_1/2_ for cT_FH_ reactive to the latter peptide was 227 days.

The most common SARS-CoV-2-reactive CD4^+^ memory phenotypes are T_CM_ and T_EM_, whose frequencies are relatively stable throughout 8 months of convalescence with negligible presence of T_EMRA_ (64, 65, 70). Similar to CD8^+^ T-cell memory, the number of CD4^+^ T lymphocytes with a T_SCM_ phenotype progressively increases for 4 months to achieve a stable plateau thereafter (70). Virus-specific CD4^+^ T_RM_ can be identified in the lungs (74).

SARS-CoV-2 induces reactive cytotoxic CD4^+^ T cells (CD4-CTLs) in COVID-19 patients (77, 78). These cells lose their co-stimulatory molecule CD28 and express perforin and granzyme B (79). Indeed, virus-specific CD4-CTLs are present in the peripheral blood, and their numbers increase in the lungs and draining lymph nodes with disease progression (77).

## 4 Preformed cross-reactive antiviral adaptive immunity

About 20-60% of COVID-19-naïve individuals have circulating CD4^+^ T cells that can cross-recognize SARS-CoV-2 spike and nonspike antigens, what brings important implications for SARS-CoV-2 primary infection and vaccination (73, 80). Most human beings have serological evidence of previous infection with endemic cold coronaviruses (HCoVs: 229E, OC43, NL63, and HKU1) (81). Moreover, a recent HCoV exposure is associated with a milder COVID-19 outcome after SARS-CoV-2 infection (82). It is intuitive to associate the phylogenetic vicinity between HCoVs and SARS-CoV-2 to the observed immune cross-reactivity (83). In support of this reasoning, a recent empirical estimate posits a 57% chance of cross-reactivity targeting sequences that share >67% homology (18). Nevertheless, what triggers this “promiscuous” immune activity is not so easily identifiable. Indeed, Tan et al. showed that more than half of the reported preexisting reactivities target epitopes in SARS-CoV-2 that do not have sequence homology with the four endemic HCoVs (84). Regardless of the existence of additional sources for antigenic priming, cross-reactive memory T lymphocytes do exist in healthy individuals and have been characterized at the epitope level. Thus, preexisting CD4^+^ T lymphocytes specific to the immunodominant S_816-830_ spike epitope are identifiable in 20% of the COVID-19-naïve subjects. Also, T cells with this specificity are recruited – with secondary reaction kinetics – in the primary immune response to SARS-CoV-2 infection in 50-60% of the cases, as well as in the reaction to the first dose of a COVID-19 vaccine in almost all recipients (85). Similarly, dominant preexisting T-cell cross-reactivities against SARS-CoV-2 targeting the nucleoprotein as well as discrete NSP7 and NSP13 epitopes have been described in half of healthy subjects (86). Other non-structural SARS-COV-2 antigen that was shown to be frequently recognized by preexisting memory CD4^+^ T cells is the highly conserved NSP12 which provides essential RNA polymerase activity to coronaviruses (47). Once initiated, T-cell expansion occurs largely transiently against the above antigen examples, to be quicky taken over in the course of COVID-19 immune response by other anti-SARS-CoV-2 specificities – perhaps in just enough time to pave the way to asymptomatic or mild outcomes (85).

Reports that describe preexisting memory CD8^+^ T lymphocytes that are cross-reactive to SARS-CoV-2 antigens are not as common as for CD4^+^ cells, and usually identify low frequency populations (47, 73, 86). One of the reasons for the failure in identifying memory CD8^+^ T cells so far may be the focus on the peripheral blood. Niessl et al. overcame this bias and used intracellular staining of 4-1BB and IFN-γ to identify tissue-resident CD69^+^ CD103^+^ CD8^+^ T lymphocytes in oropharyngeal samples collected before the onset of the current pandemic (73). They found multiple CD8^+^ SARS-CoV-2 reactivities (spike, nucleoprotein, membrane/envelope glycoproteins, ORF1a, ORF1b and ORF3-10) with lower frequencies as compared to EBV-specific T cells but still readily detectable in 32% of the individuals. Interestingly, the frequencies of tonsillar and peripheral blood CD4^+^ T cells were similar, in contrast to CD8^+^ cells which were virtually absent from the blood (73). Thus, it is conceivable that cross-reactive T_RM_ lymphocytes act as a first line of defense in the upper airway track against primary SARS-CoV-2 infection. In fact, T_RM_ was shown to confer near sterilizing immunity in murine mucosae, which is mediated by INF-γ and TNF-α, leading to major microenvironmental changes, including NK cell activation, B-cell recruitment, and local DC maturation (87).

All mentions of T-cell recognition found throughout this text refer to antigenic targets encoded by the ancestral Wuhan Hu-1 virus sequence by default unless otherwise specified.

## 5 Vaccine platforms

**Figure 1:**
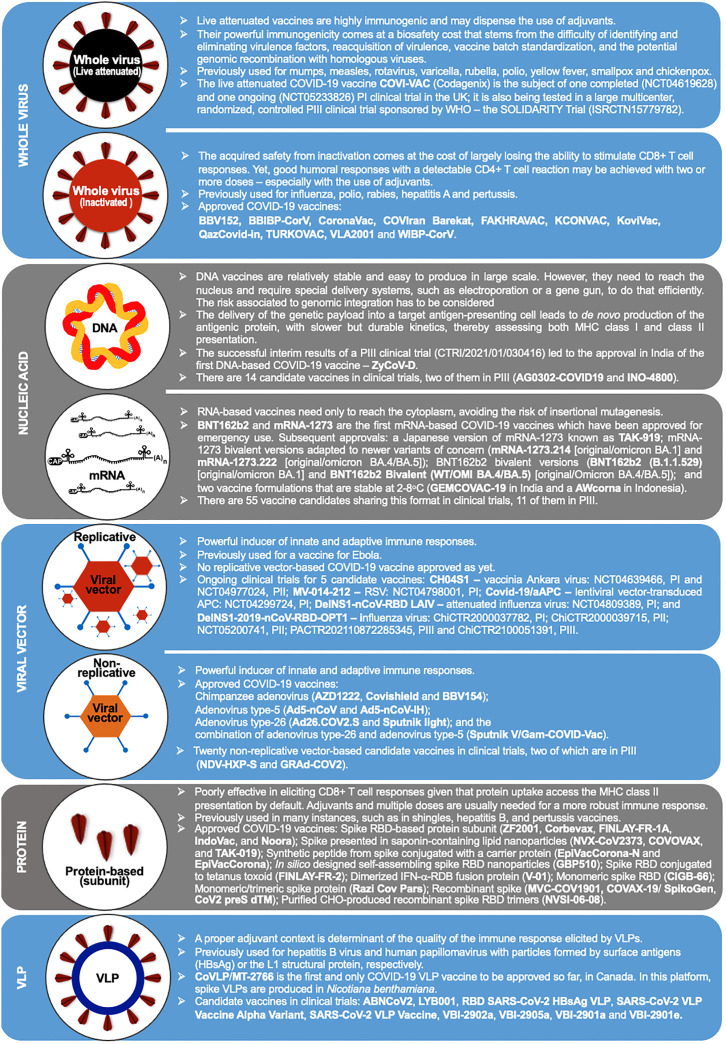
Vaccine production platforms characteristics. This figure presents a general outline of the major COVID-19 vaccines. It is complemented by **Table 2** that compiles additional information on the approved vaccines. Figure references: Live attenuated whole virus (89–93); Inactivated viral vaccines (11, 89, 94–103); DNA-based vaccines (88, 104–108); RNA-based vaccines (9, 10, 88, 109–121); Replicative viral vector-based vaccines (88, 122–125); Non-replicative viral vector-based vaccines (3, 5, 88, 126–132); Protein-based vaccines (88, 123, 133–146); Virus-like particles (88, 147–151). P1, PII, and PIII: phase I, II and III.

The immunogenic cargo of a vaccine may be carried in multiple ways. Such a diversity accounts for the 242 vaccine candidates against COVID-19 that have been tested, from which 92 completed or are undergoing phase III clinical trials (88). Five major platforms have been used for the production of the 50 COVID-19 vaccines already approved in 201 countries (**Figure 1** and **Table 2**): **(i) *Whole virus vaccines*** have reduced virulence but preserved viability and immunogenicity in the live-attenuated format. Instead, if *inactivated*, the pathogens are treated by chemical or physical means that disable their infectiousness and replicative potential (89, 94); **(ii) *Nucleic acid-based vaccines*** encode the immunogen of interest in a plasmid DNA or mRNA format. **(iii) *Viral vector-based vaccines*** carry a transgene encoding the immunogenic protein. *Replicative vectors* amplify their genomic copies and produce secondary viral particles upon infection (122). *Non-replicative vectors* retain their infectiousness but do not have the genomic information needed to produce new viral particles (157). Adenoviral vectors are by far the most archetypal members of the latter class. Pre-existing immunity generated against common human adenovirus serotypes (e.g., Ad5) and/or produced in the context of homologous vaccine re-dosing represents an important concern (3, 158); **(iv) *Protein-based vaccines*** do not have the potential biohazard associated to the genome of the original pathogen nor of a viral vector. They may be produced in large scale as protein subunits by standard recombinant technologies (123, 133); **(v)** ***Virus-like particles (VLPs) *** are self-assembling nanostructures formed by the symmetrical arrangement of natural or recombinant viral structural proteins, or even synthetic molecules, which lack a genome and cannot replicate (147). There is one approved COVID-19 vaccine and 8 other candidates in 15 clinical trials, as well as many more in the preclinical phase (88, 149). Among those, we have recently reported the development of Moloney murine leukemia virus (MLV)-like particles pseudotyped with a codon-optimized version of the spike protein (159). Protection from symptomatic disease by vaccines with the largest population coverage at the time of first authorized use is presented in **Table 1**.

**Table 2:** Approved COVID-19 vaccines.

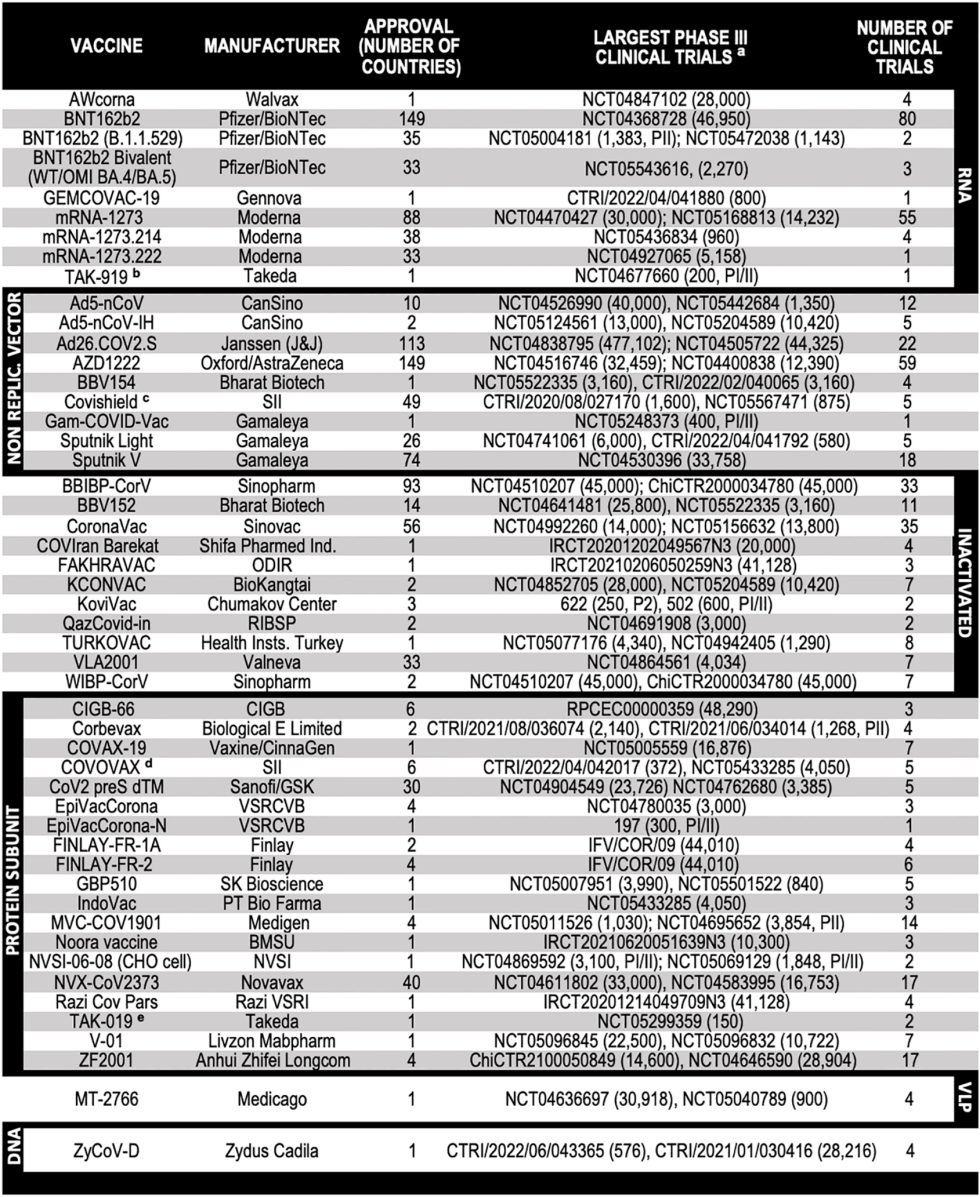	

**a:** The actual or estimated enrollment size is indicated in parenthesis. In the absence of phase III (PIII) clinical trials, phase I/II (PI/II) studies are indicated.

**b:** TAK-919 (Moderna formulation).

**c:** Covishield (Oxford/AstraZeneca formulation).

**d:** COVOVAX (Novavax formulation).

**e:** TAK-019 (Novavax formulation).

Alternative names for the vaccines listed above are: mRNA-1273 (Spikevax, Elasomeran); mRNA-1273.214 (Spikevax Bivalent Original/Omicron BA.1); mRNA-1273.222 (Spikevax Bivalent Original/Omicron BA.4/BA.5); BNT162b2 (Comirnaty, Tozinameran); BNT162b2 (B.1.1.529)(Comirnaty Bivalent Original/Omicron BA.1); BNT162b2 Bivalent (WT/OMI BA.4/BA.5)(Comirnaty Bivalent Original/Omicron BA.4/BA.5); AZD1222 (Vaxzevria, ChAdOx1 nCoV-19); Sputnik V (Gam-COVID-Vac); Ad26.COV2.S (Jcovden, Ad26COVS1, JNJ-78436735); Ad5-nCoV (Convidecia); BBV154 (iNCOVACC); BBV152 (Covaxin); BBIBP-CorV (Vero Cells)(Covilo); KCONVAC (KconecaVac); FAKHRAVAC (MIVAC); Turkovac (ERUCOV-VAC); QazVac (QazCovid-in); ZF2001 (Zifivax, RBD-Dimer); BECOV2A (Corbevax); CIGB-66 (Abdala); FINLAY-FR-2 (Soberana 02, Pastu Covac); FINLAY-FR-1A (Soberana Plus); NVX-CoV2373 (Nuvaxovid); GBP510 (SKYCovione); COVAX-19 (SpikoGen); EpiVacCorona-N (Aurora-CoV); CoVLP (MT-2766, Covifenz, Plant-based VLP); Gam-COVID-Vac (Sputnik, rAd5); and CoV2 preS dTM (VidPrevtyn Beta, SP/GSK subunit B.1.351).

NVSI: National Vaccine and Serum Institute; Razi VSRI: Razi Vaccine and Serum Research Institute; SII: Serum Institute of India; VSRCVB: Vector State Research Center of Virology and Biotechnology; CIGB: Center for Genetic Engineering and Biotechnology; Finlay: Instituto Finlay de Vacunas Cuba; BMSU: Baqiyatallah University of Medical Sciences; RIBSP: Research Institute for Biological Safety Problems; BioKangtai: Shenzhen Kangtai Biological Products; and ODIR: Organization of Defensive Innovation and Research.

The table was compiled from data available at https://covid19.trackvaccines.org (88). Published references to the cited clinical trials are: Inactivated whole virus vaccines (11, 97–103); ZyCoV-D (105, 106); mRNA-1273 (10, 114); TAK-919 (118); mRNA-1273.214 (120); mRNA-1273.222 (119); BNT162b2 (9, 115); BNT162b2 Bivalent (119); AWcorna (152); AZD1222 (3, 5); Ad5-nCoV (127); Ad26.COV2.S (129); Sputnik Light (128); Sputnik V (130); Spike RBD-based protein subunit vaccines (ZF2001, Corbevax, FINLAY-FR-1A, IndoVac, and Noora) (153–156); Spike presented in lipid nanoparticles (NVX-CoV2373, COVOVAX, and TAK-019) (136, 137); In silico designed self-assembling spike RBD nanoparticles (GBP510) (138); Spike RBD conjugated to tetanus toxoid (FINLAY-FR-2) (139); dimerized IFN-α-RDB fusion protein (V-01) (140); Monomeric spike RBD vaccines (CIGB-66) (141); Recombinant spike vaccines (MVC-COV1901, COVAX-19/SpikoGen and CoV2 preS dTM) (142–144); Purified CHO-produced recombinant spike RBD trimers (NVSI-06-08) (145) and CoVLP/Covifenz (151).

### 5.1 Post-vaccinal immunity

Two major strategies used to enhance vaccine immunogenicity are modulation of antigen pharmacokinetics and PRR stimulation. The former is commonly achieved by the use of aluminium salts to create an “antigen depot effect” that implements the slow release and long-term immune stimulation (91). The inactivated whole-virion SARS-CoV-2 vaccine CoronaVac uses alum in its formulation (94). In addition, other COVID-19 vaccines in current use or in pre-clinical testing boost their immunogenicity by their own intrinsic properties and/or by adjuvants that mimic PRR ligands in natural infection. Thus, the inactivated whole SARS-CoV-2 vaccine Covaxin uses alum gel and the TLR 7/8 agonist imidazoquinoline in its formulation (94, 160). mRNA vaccines display an intrinsic adjuvant activity because they trigger TLR-7, TLR-8 and MyD88 in a single-stranded format, as well as RIG-I and MDA5 as a double-stranded molecule, generating a robust inflammatory response (161). The COVID-19 mRNA vaccines BNT162b2 and mRNA-1273 have been modified to dampen this self-adjuvant effect in order to increase their stability and translation efficiency (162). Nevertheless, they remain a clear target for recognition by MDA5, thereby inducing the production of IFN-α and the subsequent auto/paracrine activation of IFNAR signaling, as it has been demonstrated for BNT162b2 (163). Similarly, the adenovirus vector-based vaccines ChAdOx1 and Ad26.COV2.S have an intrinsic adjuvant effect upon uptake by DCs in injection sites and lymph nodes as their double-stranded DNA triggers TLR-9 and the type-I IFN response (164). Moreover, a number of experimental vaccine candidates exhibit the dual potential of delivering the specific immunogen and being their own adjuvant (e.g., the NDV-HXP-S and GRAd-COV2) (131, 132). In addition, when administered in the proper adjuvant setting, VLPs may be strong PRR activators in DCs (165).

#### 5.1.1 mRNA vaccines

Most studies show that mRNA-1273 and BNT162b2 mRNA vaccines elicit a spike-specific CD8^+^ T-cell response in 70-90% of the immunized individuals a few weeks after the second dose, and memory cells are detectable in 41-65% of the cases seven months after the first shot (34). Accordingly, detection of CD8^+^ T cells by AIM or intracellular cytokine staining was reported in 88% of the vaccinees during the peak of the response for mRNA-1273 (166). Similar data was obtained for both BNT162b2 and mRNA-1273 (167). Moreover, by focusing on three spike-derived MHC class I-restricted T-cell epitopes that are poorly shared with other coronaviruses, Oberhardt et al. could show a response as early as 6-8 days post-prime (168). On the other side of the chronological scale, Kuse et al. were able to follow two additional spike epitopes for over 30 weeks after immunization with the BNT162b2 mRNA vaccine, attesting to the stability of the long-term CD8^+^ T cell memory to at least these immunodominant reactivities (169). The virus-specific CD8^+^ T-cell decay kinetics for both vaccines is slow, with an observed two-fold drop over the following six months (170). In fact, Goel et al. reported a contraction t_1/2_ of 27 days from peak to 3 months post-vaccination. Thereafter, they found that CD8^+^ T lymphocytes continue to decline so that they are detected in only 41% of the subjects at 6 months (167). The magnitude of the response is illustrated by the findings reported by Liu et al. on BNT162b2 vaccinees, which revealed that spike-specific lymphocytes in the peripheral blood amount to 0.028% of the total CD8^+^ T-cell response as measured by intracellular IFN-γ staining eight months after the second immunization (121).

Phenotypically, mRNA vaccination-induced CD8^+^ T cells are polyfunctional and recognize a diverse set of epitopes in the spike protein, including that from more immune evasive variants like omicron (121, 166). After priming, these cells become highly activated and proliferative as indicated by the presence of CD38 and Ki-67, and they acquire higher expression of PD-1, TOX, T-BET and CD39 after boost (168). Most cells produce IFN-γ and granzyme B and have an effector memory phenotype (170). Although the general functional profiles of the elicited CD8^+^ cellular responses to the mRNA-1273 and BNT162b2 mRNA vaccines are rather similar, Zhang et al. have observed that the T-cell frequency and polyfunctionality elicited by the mRNA-1273 vaccine tend to be higher, with more than 10% of the cells expressing simultaneously IFN-γ, granzyme B, TNF-α and IL-2 at six months after the first immunization – a pattern that is nearly absent for the BNT162b2 vaccine (170).

Virtually 100% of the individuals who receive mRNA-1273 or BNT162b2 mRNA vaccines generate circulating anti-spike CD4^+^ T cells that remain detectable at 6 months after the second dose (34). Thus, Mateus et al. showed that mRNA-1273 immunization produces spike-reactive OX40^+^ CD137^+^ CD4^+^ T cells in 97% of the individuals already after the first dose – a finding that is extended to all vaccinees after the second dose (166). It is worth pointing out that these investigators also found preexisting circulating spike-cross-reactive CD4^+^ T lymphocytes in 49% of the cases. Although the CD4^+^ T-cell response induced by BNT162b2 exhibits the same profile, its peak frequency and its magnitude at 6 months were shown, respectively, to be 1.5 and 1.8 times lower as compared to mRNA-1273 by using the AIM assay (170). When analyzed by the ICS assay, these values were 2.5- and 2.6-fold lower, respectively (170). The reported t_1/2_ of induced spike-specific CD4^+^ T cells was shown to be 47 days from peak to 3 months post-vaccination (167). Then, the decay kinetics stabilize achieving a t_1/2_ of 187 days within the 3-6-month window after immunization (167). Spike-specific lymphocytes in the peripheral blood amount to 0.033% of the total CD4^+^ T-cell response as measured by intracellular IFN-γ staining eight months after the second immunization (121).

The patterns of polyfunctionality of the CD4^+^ T-cell response induced by mRNA-1273 or BNT162b2 are rather similar with about 40% of the spike-specific lymphocytes being capable of producing simultaneously 2-4 effector molecules (IFN-γ, IL-2, TNF-α, and granzyme B) six months after the immunization as reported by Zhang et al. (170). Nevertheless, these investigators found that mRNA-1273 produced higher frequencies of cells that stained positive: (i) for either TNF-α or IL-2 early on and at 6 months after the first dose and (ii) for IFN-γ at 6 months after the first immunization (170). Moreover, besides the cytokine secretion profile commonly associated with Th1 polarization, spike-specific CD4^+^ T lymphocytes with the cT_FH_ phenotype were detected in 75% of the vaccinees after the second dose of mRNA-1273 and remained detectable in 63% of the cases over 6 months thereafter (166). Similar data also appeared in a vaccine head-to-head comparison which did not show much difference, with spike-specific cT_FH_ cells found in 97% and 81% of the mRNA-1273 and BNT162b2 vaccinees after the second dose, respectively, remaining detectable in >80% of these subjects 6 months post-vaccination (170). The predominant mRNA vaccine-induced spike-specific memory phenotypes were shown to be T_CM_ and T_EM_ at peak. T_CM_ frequency largely declines during the post-peak contraction but T_EM_ stabilizes during the 3-6-month post-vaccination window (167). In addition, the two mRNA COVID-19 vaccines also induce cytotoxic CD4^+^ T cells (CD4-CTL). These are terminally differentiated lymphocytes that may be found as oligoclonal populations in the response to other types of chronic antigenic stimulation (79). Indeed, the circulating granzyme B^+^ CD4^+^ T-cell number increases after the first and second vaccine doses, reaching a plateau at 3.5 months after the first dose which remains stable for the following 2.5 months (170).

#### 5.1.2 Adenoviral vector-based vaccines

Two doses of 5 x 10^10^ viral particles (vp) of the AZD1222 vaccine were shown to trigger a spike-specific CD8^+^ T-cell response with a frequency of 0.03% at day 56 (4 weeks after the second immunization), which was mostly composed of IFN-γ-producing lymphocytes (171). In addition, most of these cells were polyfunctional, with 23-32% of them simultaneously producing TNF-α, IL-2 and IFN-γ. The spike-specific T-cell receptor (TCR) repertoire of the CD8^+^ subset was also studied in AZD1222 vaccinees revealing diversity of unique sequences (breadth) as well as increased frequency (depth) on day 28 after the second dose, albeit at lower levels than those observed in Th1 CD4^+^ T lymphocytes (171). Although half of the TCRs of CD8^+^ T cells recognized the region corresponding to amino acids 265-277, the remainder recognized epitopes were distributed throughout the entire spike protein (171).

Individuals immunized with AZD1222 already exhibit a clear CD4^+^ Th1 bias after a single dose (172). Understandably, a robust anti-spike CD4^+^ Th1 response appears after 2 administrations of this vaccine as measured by intracellular cytokine staining of PBMCs stimulated with spike peptides *in vitro* (171). Thus, the median frequency of CD4^+^ T lymphocytes reaches 0.062% at day 56 but remains lower than the corresponding frequency found in convalescent COVID-19 patients (0.13% in this study). Differently from the CD8^+^ response, most spike-specific CD4^+^ T cells produce TNF-α (0.06%), as well as IL-2 (0.04%) and IFN-γ (0.03%) (171). Two doses of the vaccine produced a TCR repertoire in the CD4^+^ subset capable of recognizing epitopes throughout the spike protein with comparable depth and breadth found in convalescent COVID-19 patients (171).

A single dose of 5 × 10^10^ Ad26.COV2.S vp was shown to elicit a spike-specific CD8^+^ T-cell response in 51% and 36% of immunized individuals aged 18-55 and 65 or older, respectively (173). This CD8^+^ T-cell response is durable for at least eight months as demonstrated by two studies: one that reported frequency magnitudes of 0.036% and 0.061% at four weeks and at eight months after the immunization, respectively (121), as well as another that registered the same frequency magnitude of 0.12% at both time points (174). Similar results were reported later with 67% of the individuals showing a detectable CD8^+^ T-cell response at 15 days and 64% at 185 days post-vaccination (170). The same study also revealed that IFN-γ is the predominantly produced cytokine by CD8^+^ T lymphocytes upon restimulation with a spike peptide megapool, and more than 70% of these cells remain polyfunctional, often coexpressing IFN-γ and granzyme B and, less frequently, TNF-α. As for the breadth and depth of the Ad26.COV2.S-induced spike-specific TCR repertoire of the CD8^+^ subset, there is substantial induction of moieties capable of recognizing epitopes throughout the spike protein (175).

Most individuals (71%-100%) mount a spike-specific CD4^+^ T-cell response to Ad26.COV2.S, which remains detectable by the AIM assay over 6 months post-vaccination (170). However, the peak magnitude is about 2- and 3-fold lower than that elicited by BNT162b2 and mRNA-1273, respectively (170). The Ad26.COV2.S vaccine generates a Th1-skewed response detectable in 60%-76% of the recipient subjects by the ICS assay, with people 65 years of age or older being the least responsive (173). The reactive lymphocytes reach a median frequency of 0.043% at peak but descend to 0.018% at 8 months post-vaccination (174). Zhang et al. reported a more stable long-term kinetics for the spike-specific CD4^+^ T-cell response to Ad26.COV2.S, with a peak achieved at 2 weeks followed by the establishment of a plateau during the 6 months after the immunization (170). Sequencing of the TCR β chain of spike-specific CD4^+^ T cells revealed a considerable number of unique rearrangements (175). About one third of these lymphocytes are polyfunctional being capable of producing 2 or 3 effector molecules (170, 175). The most frequent secretory pattern was TNF-α only, trailed by the following combinations: (i) TNF-α + IL-2, (ii) TNF-α + IFN-γ + IL-2, and (iii) TNF-α + IFN-γ (175). Ad26.COV2.S was also shown to generate spike-specific CD4^+^ cT_FH_ in 71%-79% of the vaccinees. These cells reach a circulating frequency peak at 2 weeks post-vaccination and remain at this level for 6 months (170).

#### 5.1.3 Inactivated whole-virion vaccines

The stimulation of SARS-CoV-2-specific cytotoxic T lymphocytes by whole-virion vaccines is the subject of some debate (176). Yet, a recent report showed that CoronaVac induces a CD8^+^ T-cell response to either spike, nucleoprotein or membrane glycoprotein in 58-65% of vaccinees 4 weeks after the second dose as measured by intracellular cytokine staining (177). The IFN-γ^+^ CD8^+^ T lymphocyte frequencies in PBMCs were 0.015% and 0.041% for spike and combined reactivities (spike, nucleoprotein and membrane glycoprotein), respectively. IL-2-producing cells were also detected but at lower frequencies. A separate study followed-up the kinetics of the CD8^+^ T-cell memory immune response to CoronaVac vaccination over one year: the relative percentage of virus-reactive CD8^+^ T_EM_ cells were 9.48%, 12.14%, 5.73% and 0.89% at 1, 3, 6 and 12 months, respectively (178). Conversely, the T_EMRA_ subset increased from almost undetectable early-on to 8.74% at 12 months. These memory cells exhibit different cytokine production kinetics: IFN-γ, granzyme B and IL-2 peak at 3-6 months, 1 month and 6-12 months, respectively. It is noteworthy that the CD8^+^ memory T cells are still reactive upon restimulation being capable of producing the three cytokines one year after immunization (178).

Duque et al. reported that CoronaVac triggers a robust spike- and SARS-CoV-2-specific CD4^+^ T-cell response in about 77-83% of the vaccinees during the 4 weeks that follow the second dose (177). The IFN-γ^+^ T lymphocyte frequency reached 0.068% after 2 doses as measured by the combined reactivities to spike, nucleoprotein and membrane glycoprotein (177). Also, Zhao et al. analyzed the anti-viral CD4^+^ T-cell response in CoronaVac vaccinees for a longer period and found that T_EM_ cells start from negligible frequencies at 1 month to become readily detectable at 12 months post-vaccination (178). Conversely, these authors showed that anti-viral T_CM_ accounts for about 11%-15% of CD4^+^ T cells during the first 3 months post-vaccination, dwindling thereafter to reach close to 1% at 12 months (178). Importantly, by the end of this analysis (one-year post-vaccination), both T_EM_ and T_CM_ retained their reactivity upon *in vitro* stimulation, with higher frequency of antiviral lymphocytes capable of producing IL-2, as well as lower but detectable numbers of cells that produce IFN-γ and granzyme B as compared to the first 3 months post-vaccination (178). Data from the PROFISCOV clinical trial indicate that CoronaVac produces a CD4^+^ T cell response in the vaccinees that is primarily directed at spike and that is sustained for at least 6 months. The reactivities to other viral products are better identified only later in the post-vaccinal course (3-6 months). Importantly, the investigators failed to identify any CD8^+^ T cell reactivity in the vaccinees by using an AIM assay (179).

Two doses of the BBV152/Covaxin induced a CD8^+^ T-cell response that could be detected in peptide megapool-stimulated PBMCs by the AIM assay in 15/30 vaccinees as spike-specific and in 10/24 vaccinees as nucleoprotein-specific (160). A separate study found that T_EMRA_ was the major memory CD8^+^ subset (13.7%) identified in 8 vaccinated individuals at six months after the second dose (180). In addition, BBV152/Covaxin generated a robust CD4^+^ T-cell response in most individuals (85%), which was stable for 6 months and included cells capable of recognizing spike and nucleoprotein with a frequency comparable to infection (160). These lymphocytes were Th1-skewed with production of TNF-α, IL-2 and IFN-γ. The two largest CD4^+^ memory subsets were: T_EM_ that contracted and T_CM_ that expanded over 200 days post-vaccination. From the total CD4^+^ T lymphocytes, 0.11% corresponded to spike-specific and 0.07% to nucleoprotein-specific cT_FH_ (160).

It should be pointed out that another discordant voice came from Lim et al. who described the virtual absence of a CD8^+^ T-cell response to inactivated vaccines (176). Instead, they show in their study arm that received 2 doses of BBIBP-CorV that this vaccine produces a robust CD4^+^ T-cell response, comparable in magnitude to that induced by mRNA immunization but with higher breadth as it also encompasses antigenic targets other than spike (nucleoprotein and membrane glycoprotein). Importantly, there was no major waning of the CD4^+^ T-cell response over 6 months post-vaccination (176). Those authors attribute their finding of exclusive stimulation of CD4^+^ T lymphocytes by inactivated vaccines to differences in analytical methods. In their rigorous experiments, they have depleted CD4^+^ T cells before examining the CD8^+^ T-cell reactivity to virus peptide pools and vice versa, thereby eliminating any potential antigen-independent bystander activation.

#### 5.1.4 Recombinant protein vaccines

NVX-CoV2373 is a nanoparticle vaccine built with recombinant SARS-CoV-2 full-length spike (181). Although purified or recombinant proteins may have limited intrinsic immunogenicity for T cells, particularly for the CD8^+^ subset, Moderbacher et al. managed to identify a modest spike-specific CD8^+^ reactivity in 20%-26% of NVX-CoV2373 vaccinees one week after the second dose by ICS/AIM assays (182). Most of these lymphocytes produced IFN-γ and some of them produced a combination of the following cytokine/effector molecules: IFN-γ, granzyme B, IL-2 and TNF-α. This finding might be a consequence of the vaccine saponin-containing adjuvant that facilitates cross-presentation (183). In other study with a longer follow-up, the anti-spike CD8^+^ T-cell response rate as measured by the AIM assay (CD69^+^ CD137^+^) achieved 70% and 80% of the vaccinees at 3.5 and 6 months, respectively (170). This response was also detectable by a modified ICS assay (as defined as cytokine^+^ CD69^+^ CD8^+^ peripheral lymphocytes) in 10% of the subjects at 3.5 months after the first immunization, which increased to 50% at 6 months. However, the circulating CD8^+^ T lymphocytes elicited by NVX-CoV2373 were less abundant as compared to other vaccine platforms and were mostly capable of producing IFN-γ upon *in vitro* stimulation with spike peptide megapools at frequencies comparable to convalescent patients at 6 months (170).

The majority of the NVX-CoV2373 vaccinated individuals (81%) mounted a spike-specific Th1 CD4^+^ T-cell response one week after the second dose, with 35% of the lymphocytes being capable of simultaneous production of 3-5 effector molecules (IFN-γ, IL-2, TNF-α, iCD40L and granzyme B) (182). A fraction of the CD4^+^ cells also exhibited CXCR5 expression and were detectable as cT_FH_ in 44% of the vaccinees after the second dose (182). Similar findings by other investigators indicate that virtually all NVX-CoV2373 vaccinees had circulating CD4^+^ T cells with Th1 and cT_FH_ phenotypes by the AIM assay. Intracellular staining revealed that close to 40% of the spike-reactive lymphocytes were polyfunctional as well as that CD4-CTLs were present in 80% of the cases at 6 months after the first immunization (170).

A direct comparison of the approved vaccines as regards the elicited T cell response would be unfair because there are very few studies that perform side-by-side comparisons and the predominant focus on B cell responses as a correlate of efficacy. Moreover, such studies are relatively small and, most importantly, do not exhibit methodological analytical uniformity. With this caveat in mind, **Table 3** presents some of the characteristics of the vaccine-induced anti-viral T cell response stratified by production platform.

**Table 3:** T cell response to COVID-19 vaccines stratified by production platform.

Vaccine Platform		CD4/CD8	Responders (%)	Memory persistence (months)	Magnitude ^a^ (% of total subset)	Targeted Viral antigen ^b^	Polyfunction	CD4 helper subsets
mRNA	(BNT162b2, mRNA-1273)	CD8	70-90	7-8	0.012-0.028	S	+	
		CD4	~100	6-8	0.054-0.14	S	+	TH1, cTFH
Adenoviral	(AZD1222, Ad26.COV2.S)	CD8	36-67	8	0.031-0.12	S	+	
		CD4	71-100	8	0.017-0.026 ^c^	S	+	TH1, cTFH
Inactivated whole-virus	(CoronaVac, BBV152, BBIBP-CorV)	CD8	0-65 ^d^	6-12	0.041 ^e^	S, N, M	+	
		CD4	77-85	6-12	0.068 ^e^	S, N, M	+	Th1, cTFH
Recombinant	(NVX-CoV2373)	CD8	20-80	6	0.018	S	-/+ ^f^	
		CD4	81-100	6	0.097	S	+	Th1, cTFH

a: Fraction of the CD4^+^ or CD8^+^ T cell compartments that recognizes spike or the combined spike, nucleoprotein and membrane glycoprotein antigens by intracellular cytokine staining (IFN-γ for CD8^+^ T cells and IFN-γ, TNF-α, IL-2 or granzyme B for CD4^+^ T lymphocytes). The measurements were made at 6-8 months post-vaccination or as otherwise indicated. Please consider these numbers as an illustration only because real-world values may vary wildly.

b: S (spike), N (nucleoprotein) and M (membrane glycoprotein).

c: The value 0.026% refers to IFN-γ^+^ CD4^+^ T cells alone.

d: Although a CD8^+^ T-cell response to inactivated whole-virus vaccines have been identified by the AIM assay, other investigators failed to do so as discussed in section 5.1.3.

e: Measurement made 4 weeks after the second dose; and.

f: Although Moderbacher et al. had identified anti-spike CD8^+^ T cell polyfunctionality 1 week after dose 2, Zhang et al. identified mostly IFN-γ-producing T lymphocytes at 6 months post-vaccination (170, 182). The table was based on this article and on references 34, 121, 166, 170–174, 176, 177, and 182.

## 6 Multivalent vaccines

The emergence of SARS-CoV-2 VOCs with immune evasive capacity has prompted the World Health Organization (WHO) to create the Technical Advisory Group on COVID-19 Vaccine Composition (TAG-CO-VAC), which advocates global access to current vaccines and envisage the antigenic updating of newer versions (184). Several multivalent vaccines are being tested, including those that are produced as recombinant proteins with: alfa and beta (185); beta and delta (186); and beta, kappa, and the prototypical (145) spike amino acid sequences. As for the bivalent mRNA formulations, the ancestral Wuhan-Hu-1 spike coding sequence is carried alongside: the beta (187); the BA.1 (120, 188); or the BA.4/BA.5 (189–191) corresponding sequences. The latter two formulations have been approved by regulatory agencies in several countries (**Table 2**). There is not much information about the impact of the multivalent format of the above vaccines on the elicited antiviral T-cell response. Yet, data analysis covering over 360,000 nucleic acid amplification tests performed during a period of omicron prevalence revealed that those individuals who got 2-4 doses of the monovalent mRNA vaccine followed by the BA.5-encoding bivalent booster had higher protection from SARS-CoV-2 symptomatic infection as compared to unvaccinated people (189). The same report found that bivalent vaccines conferred a modest additional protection when the comparison was made with individuals who only received 2-4 doses of the monovalent mRNA preparation (189). These results were corroborated by another study that has identified a lower risk of emergency care/hospitalization among those who received the BA.5-containing bivalent immunization (190). For emergency care encounters, the bivalent absolute vaccine effectiveness was 56% against no vaccination and the relative effectiveness was 32% for those who had previously got 2-4 monovalent shots – the last of which being 2-4 months earlier. Interestingly, the relative effectiveness of the bivalent shot increased to 50% if the interval since the last monovalent dose was ≥ 11 months. The waning of the monovalent immunization makes the relative effectiveness of the bivalent shot to be higher with longer intervals (190). Similar protection levels were achieved for hospitalization.

Viral evolution follows a fast pace, so that by the time the first bivalent vaccines gained regulatory approval and were deployed, the subvariant landscape had already changed. Thus, their true protective efficacy relies, at least partially, on their breadth and immune cross-reactivity against more evasive VOCs. Indeed, an early estimate made by the Centers for Disease Control and Prevention (CDC) of the vaccine efficiency of the BA.5-encoding mRNA preparation used as a booster dose indicates that it provides additional protection against symptomatic infection by omicron BA.5 itself and by the XBB/XBB.1.5 sublineages (191).

Antibody neutralization titers have been considered to be a major correlate of protection for COVID-19 vaccines (192). Indeed, when used as a booster fourth dose, the BA.5-encoding bivalent vaccine is generally more efficient in eliciting neutralizing antibodies against BA.2 and BA.5 derivatives than the original monovalent version as illustrated by Zou et al. (193). However, the absolute titers tend to be rather low against the most evasive variants: The bivalent booster-induced anti-XBB.1 neutralizing activity in the previous example was close to 40 times lower than that against the ancestral virus (193). In line with this view, sera from fully immunized individuals with 3 doses of the original monovalent mRNA product who subsequently received the BA.5-encoding bivalent vaccine as a booster fourth shot virtually did not neutralize the omicron BQ.1, BQ.1.1, XBB, and XBB.1 subvariants (194). Using a similar booster protocol, other investigators also observed low neutralization activity against the BA.2.75.2, BQ.1.1 or XBB.1 sublineages (195, 196). The Coronavirus Variant Immunologic Landscape Trial (COVAIL) is the first randomized clinical trial to compare head-to-head the two approved bivalent vaccines used as a booster. Its preliminary findings revealed poor induction of antibody neutralization of the BQ.1.1 and XBB.1 subvariants with titers 13-35 times lower (for the BA.1-containing bivalent vaccine) and 8-22 times lower (for the BA.4/BA.5-containing bivalent vaccine) as compared to those generated against the Wuhan-1 variant carrying only the D614G spike mutation (197). Another recent report indicates that sera from BA.4/BA.5-encoding bivalent vaccinees do show some rescued ability to recognize omicron most evasive subvariants (e.g., XBB.1 and XBB.1.5) as compared to sera from those who received 3-dose monovalent mRNA shots. Nevertheless, the titers achieved were fairly low or at the detection limit (or even under this threshold) as in the case of BQ.1.1, CH.1.1, and CA.3.1 subvariants (198). Additionally, two recent well-designed, albeit small, studies reported that a booster shot with the latter bivalent vaccine did augment the anti-SARS-CoV-2 serum neutralizing activity in previously immunized individuals but was not overtly superior to a monovalent mRNA booster in doing so (199, 200). One of these reports also provided a rare picture of the antiviral T cell response elicited by the BA.5-encoding bivalent mRNA vaccine (200). Thus, the bivalent booster was shown to increase the anti-BA.5 CD8^+^ T-cell frequency to 0.046% from a baseline of 0.024% achieved by previous triple monovalent vaccination. The corresponding frequencies for CD4^+^ T lymphocytes were 0.072% post-bivalent boosting and 0.051% at baseline. It is worth mentioning that both monovalent and bivalent boosters had comparable effect on CD8^+^ and CD4^+^ T-cell frequency (200).

The above-mentioned studies support four premisses: **(i)** Bivalent vaccines used as a booster dose are associated with increased crossprotection from symptomatic SARS-CoV-2 infection; **(ii)** The subvariants with elevated antigenic drift that currently prevail in many regions of the world (e.g., BA.5-derived BQ.1 and BQ.1.1 or the BA-2 recombination derivatives XBB and XBB.1.5) may evade neutralization by sera from vaccinees who received the bivalent booster; **(iii)** The failure to control the spread of the latter subvariants may allow the emergence of more dangerous derivatives; and **(iv)** The additional protection provided by the bivalent booster is likely to have an important T cell component.

## 7 Hybrid immunity

A relatively modest modulatory impact of previous SARS-CoV-2 infection on the magnitude of the CD8^+^ T-cell response elicited by mRNA vaccination against the ancestral virus strain has been reported (167). Indeed, the number of naïve and convalescent individuals with detectable post-immunization reaction is comparable and the magnitude of the peak of circulating spike-specific CD8^+^ T cells remain unaltered after 2 doses of either BNT162b2 or mRNA-1273 (167). Importantly, the rapid post-peak kinetics contraction observed in SARS-CoV-2 naïve mRNA vaccinees also occur with immunized COVID-19 recovered patients (167). However, as expected, the proportion of spike-specific CD8^+^ T lymphocytes versus those capable of recognizing other viral antigens increase substantially after spike-based vaccines, such as BNT162b2, are administered to COVID-19 recovered individuals (69). This finding may be interpreted as a recall expansion of infection-induced memory cells, which were shown to be biased to T_EMRA_ and to display a diverse TCR repertoire (69). In contrast, Gao et al. reported discordant results obtained with the highly sensitive spheromer technology, claiming a 3.6-54-fold size reduction alongside loss of effector function in the spike-specific CD8^+^ T-cell compartment in post-infection BNT162b2 vaccinees (201).

SARS-CoV-2 infection affects the CD4^+^ T-cell response elicited by mRNA vaccination modestly as regards detectability, memory phenotype composition, Th1 bias, generation of cT_FH_, and the magnitude at 6-8 months post-vaccination (167, 201). This pattern is also maintained even when the analysis goes to the single epitope level. Thus, single-dose immunized convalescent patients and two-dose infection-naïve vaccinees generate comparable frequencies of CD4^+^ T lymphocytes specific to the spike epitope S_751-767_, and a third booster vaccine dose did not further this frequency beyond the peak already achieved (75). Nevertheless, despite the described overlap in induced response patterns, Rodda et al. found an important qualitative difference in the CD4^+^ T-cell response to SAR-CoV-2 in hybrid immunity which is not fully captured by the antigenic exposure provided by immunization alone – the augmented frequency of IFN-γ- and IL-10-producing spike-specific cells (202). It should be added that vaccine platforms other than mRNA, such as Ad26.COV2.S, have also been evaluated and shown to impact modestly the T-cell response in the context of hybrid immunity (203).

The most remarkable consequence of hybrid immunity is the observed synergy between natural infection and vaccination in boosting binding and neutralizing antibody titers against SARS-CoV-2, which may exhibit considerable cross-reactivity against variants of concern (VOC) (15, 204, 205). Indeed, Walls et al. reported that spike-specific IgG-binding titers were about 7.5-12 times higher in hybrid immunity as compared to 2 doses of mRNA vaccines in infection-naïve individuals at peak time points (206). In addition, hybrid immunity was 10-fold more efficient when these two groups were compared for neutralization activity against a vesicular stomatitis virus (VSV) that was pseudotyped with a SARS-CoV-2 spike carrying the G614 mutation (206). These investigators have also shown that a third vaccine dose rescues the antibody titer and the *in vitro* neutralization activity to the level of hybrid immunity. In fact, triple-vaccinated individuals had retained serum neutralizing activity *in vitro* against beta, delta, and omicron variants. For omicron, the neutralization level achieved by the third dose was 11-fold lower than that observed with the G614 spike-decorated VSV, although remaining comparable to that conferred by hybrid immunity (206).

The emergence of the omicron VOC has created more convolution to an already complex field. Its major subvariant lineages (BA.1, BA.2, XE, BA.2.12, BA.2.75, XBB, BA.3, BA.4, BA.5, BQ.1 and BQ.1.1) carry up to 36 substitutions in the spike sequence and 59 mutations distributed throughout the genome, which make them highly infectious (17, 207). Breakthrough infections that were relatively rare events in the early stages of the pandemic are no longer uncommon. Although available vaccines were capable of preventing infection and reinfection by the ancestral SARS-CoV-2 strain and its first variants with great efficiency, omicron immune evasiveness proved to be a formidable challenge. Thus, two doses of the BNT162b2 mRNA vaccine that were > 90% efficient in protecting against symptomatic infection by earlier variants provide only negligible protection against omicron BA.2 at eight months or more after the second dose (208). A third dose is necessary to regain close to 50% protection (208). Also, previous SARS-CoV-2 infection alone gives only limited protection against symptomatic BA.2 reinfection (46%). Nevertheless, hybrid immunity provided by infection followed by 3 doses of BNT162b2 raises resistance to symptomatic BA.2 infection to close to 80% (208). Thus, prior infection plus vaccination (either Ad26.COV2.S or BNT162b2 or mRNA-1273) are associated with a robust spike-specific T-cell response that recognizes the original virus strain and delta in most individuals. Although the cross-reactive capacity of CD4^+^ T lymphocytes for the omicron spike is generally preserved in these subjects, about 40% of them had the omicron spike-specific recognition by the CD8^+^ T cell subset compromised, with > 50% drop in reactive proliferation (17). It should be added that the T-cell recognition of other omicron antigens (nucleocapsid/membrane/envelope/ORF3A) is preserved in hybrid immunity (17).

Of note, Lim et al. detected CD69^+^ CD103^+^ tissue resident CD4^+^ and CD8^+^ T lymphocytes in the nasal mucosa of virtually all tested breakthrough infection patients but failed to find these cells in vaccinated-only individuals (209). Interestingly, they also observed that this mucosal response was durable (remaining for at least 140 days post-infection), and exhibited a clear bias in favor of CD8^+^ T cells capable of recognizing not only spike but also nucleoprotein and NSP12 antigens (209).

Altogether, it is reasonable to attribute the better immune shielding associated to hybrid immunity to the possible combination of several factors: **(i)** the enhanced titers of virus-binding and virus-neutralizing antibodies (15); **(ii)** the breadth of the immune response that goes beyond the spike antigen commonly used in the major vaccine platforms (64, 66); and **(iii)** the generation of tissue resident T-cell populations, notably CD8^+^ T lymphocytes, that could have a central role as one of the first effective defense lines against SARS-CoV-2 reinfection in the upper respiratory tract (209).

However, one might wonder whether all hybrid immunity formats might boost the T-cell immune response in the same fashion. To address this issue, it is required to consider the immune modulatory impact of the primary infectious antigenic encounter prior to vaccination as well as the VOC genetics in case of a breakthrough infection. Thus, the hybrid immunity generated by previous Wuhan Hu-1 SARS-CoV-2 infection plus triple BNT162b2 vaccination boosts vaccine-induced T-cell reactivity against the ancestral strain and former common VOCs (e.g., delta), yet it damps omicron recognition. Also, omicron breakthrough infection in SARS-CoV-2-naïve individuals generates T cells that recognize Wuhan-Hu-1 and delta but fail in recognition of omicron itself (16). Finally, the composite scenario represented by those individuals who had five previous antigenic exposures – the first being Wuhan-Hu-1 infection, followed by three BNT162b2 vaccine doses, and a fifth being omicron breakthrough infection – abrogates subsequent omicron T-cell recognition but boosts T-cell reactivity against other VOCs (16). This paradoxical reactivity was defined as “hybrid immune damping” and, based on immunization experiments conducted with HLA transgenic mice, it was hinted that a switch to a regulatory T cell program might be at play (16).

## 8 Discussion

### 8.1 Vaccines

The first administration of a COVID-19 vaccine in humans after safety and efficacy results of a phase III clinical trial happened in December 2020 (116). The development of the mRNA technology behind BNT162b2 and mRNA-1273 was remarkable given that issues regarding stability, translation efficiency, and inflammatory overreaction had to be solved over the preceding years (109–111). The resulting prototypical vaccines have evolved from first-in-human to the deployment of hundreds of millions of doses in record time (110) (**Table 1**). In parallel, the adenoviral vector-based AZD1222 vaccine followed expeditiously a somewhat beaten path, and turn out to be successfully deployed worldwide (3, 88) (**Table 1**). Finally, the inactivated whole-virion CoronaVac and BBIBP-CorV vaccines played a major role in the control of the pandemic as they were cheaper to produce and their deployment logistics was easier to implement (94). Altogether, the above 5 vaccines account now for the bulk of the COVID-19 immunization of the world population (**Table 1**).

Does the described *status quo* warrants safe navigation for the remainder of the pandemic? Conventional wisdom seam to point otherwise. We are certainly safer – but not safe enough. In fact, the shortcomings of the vaccinal protection soon appeared with the observed fast decay of neutralizing antibodies and the emergence of VOCs with breakthrough potential. There was a sharp drop in protection efficacy against infection observed from the time when the above pioneering vaccines were granted their first emergency use authorizations to the pandemic phase in which delta – and more recently omicron – took over. This might be a direct result of the first-generation COVID-19 vaccine designs that were focused on limiting virus transmission primarily through neutralizing antibodies. Such strategy was intuitive considering that it has worked before for other viruses (89). It is worth noting that it has also failed as it did blatantly for HIV (210). Nevertheless, SARS-CoV-2 is not as genomically unstable as HIV because of the proofreading activity provided by the NSP14-10 complex (211). Yet, the VOC list keeps growing (212).

We owe to the first-generation COVID-19 vaccines the partial control of the pandemic – saving countless lives and providing some normalcy. Having acknowledged that, we may still need to adjust course to get through the end of the pandemic successfully. Indeed, unintended consequences of our previous actions may come into play. Thus, the most popularly deployed vaccines used classical and ingenious new technology to elicit biding and neutralizing antibodies against the spike protein ignoring other antigenic targets. Alternatively, the pathogen entire antigenic cargo was used in the inactivated whole-virus vaccines but in a format that is heavily biased to MHC class II presentation (95, 96). The bet was – and somehow still is – largely on eliciting anti-viral humoral responses. We believe that this bet is no longer good.

Highly infectious VOCs like omicron have produced COVID-19 surges affecting both infection-naïve and recovered individuals (208). The once-recommended standard 2-dose schedule adopted for most vaccines no longer adequately protects against omicron and its subvariants. Thus, an expert panel from the UK Health Security Agency estimates that 3 major vaccines used in that nation so far (AZD1222, mRNA-1273 and BNT162b2) only retain 20-30% efficacy against symptomatic infection by BA.1 or BA.2 omicron 4-6 months after full immunization (213). It also concludes that an mRNA booster shot is required to bring vaccine efficacy to 40-45% at 4-6 months thereafter, with complete disappearance of protection by 9 months. The rescued protection appears to depend on the booster shot choice. Indeed, Ranzani et al. reported that 2 doses of CoronaVac provided some protection (37%) against symptomatic delta infection at 6 months after the second dose but failed do so for omicron, reaching only 3.9% efficacy (214). A booster shot with BNT162b2 in these individuals raised anti-omicron protection to 33.8% but a third CoronaVac dose had no effect against symptomatic omicron infection at 2 months after its administration. This adverse scenario compels us to explore new directions. Unfortunately, the degree of long-term protection against the most evasive VOCs conferred by one of them – the bivalent booster – is yet to be tested in large-scale real-world deployment.

SARS-CoV-2 gave the impression at first that only minor adjustments in our response to the pandemic would suffice to accommodate what appeared to be an inconsequential genetic drift (215–219). Not anymore. Fortunately, though, despite the sharp drop in anti-omicron protection from infection observed after the primary 2-dose schedule and the transient partial rescue provided by booster shots, the surge in numbers of infected individuals was not accompanied by a proportional increase in severe disease and death rate (213, 214, 220–223). This is likely a fortuitous unintended consequence of the adopted vaccine strategies which also allowed the generation of T-cell responses, whose evasion is more difficult to achieve, and that compensates for the patchy humoral antiviral reaction.

The updated versions of existing vaccines as well as new candidate ones should be good elicitors of both humoral and cellular immune responses (by design and not by chance)!. Thus, we urgently need to incorporate additional antigenic targets other than spike variants into the current vaccine platforms – particularly the mRNA and adenoviral vector-based ones. In this regard, T-cell epitope-rich moieties, such as the SARS-CoV-2 nucleoprotein for which memory precursors are found after natural infection (65) or nonstructural proteins that play a crucial role early in viral infection as the viral RNA polymerase NSP12 (47), would be attractive choices. Additionally, it would be worthwhile to pay more attention to vaccine formulations. The antigen(s) choice and the vector used to deliver the antigenic cargo or its encoding information often deserve the bulk of attention. However, the formulation itself and the adjuvant choice in particular may be crucial to attain a balanced response that includes humoral and cellular components. This is especially important to enhance the immunogenicity of cheaper and logistically less-challenging alternatives, such as protein-based, inactivated whole-virion, VLPs, and DNA-based vaccines. There are innovative strategies that facilitate cross-presentation, such as the one adopted for the NVX-CoV2373 formulation, which uses amphiphilic saponins to destabilize the endosomal compartment membrane, allowing access of vaccine antigens to MHC class I presentation (182). Nevertheless, many vaccine candidates in preclinical stages still resort to centenarian alum-based recipes with little else in their formulations to enhance the cellular immune response.

### 8.2 Immune dysregulation

Several SARS-CoV-2 facets have been extensively explored in the literature, often in connection with respiratory epithelial damage and immunothrombosis (224, 225). Moreover, it is generally accepted that the virus needs to down-play the type-I IFN response to establish its infection in the host (39). Yet, it is conceivable that the viral modulatory capacity may go beyond the innate compartment. Thus, increased C3a formation by the complement activation cascade in severe COVID-19 promotes differentiation of highly activated CD16^+^ cytotoxic T cells, which may display TCR-independent, antibody-dependent cellular cytotoxicity, and lead to vascular endothelial damage (226). Similarly, the SARS-CoV-2 impact on plasmacytoid DCs, perhaps through the engagement of CD304, may not only reduce IFN secretion but have implications on the activation status, phenotypic differentiation, and composition of T-cell subsets (227, 228). Let’s also remember that antiviral T cells tend to exhibit activation markers with unusually long expression kinetics, what was even confused with an exhaustion phenotype (67, 68). In support of a possible adaptive dysregulation scenario, there is recent evidence reported by Meckiff et al. of immune phenotypic singularities in COVID-19 patients, such as the lower representation of T_REG_s as well as the increase of the CD4-CTL and cytotoxic T_FH_ subsets – especially in severe presentations of the disease (78). Additionally, these investigators identified less abundant SARS-CoV-2-specific polyfunctional Th1 cells as compared to the response to common viruses such as influenza (78). Moreover, it should be remembered that SARS-CoV-2 infection leaves an immune imprinting in the host that may affect the susceptibility to breakthrough infection in a variant-specific way (16). Thus, no one seriously know yet the full extent of SARS-CoV-2-induced immune dysregulation. It would be reasonable by analogy to have similar concerns about a putative vaccine-induced immune dysregulation – encompassing hyperactivated antiviral T cell responses – to drive post-vaccinal immunopathology. In this scenario, a single viral protein would be the likely culprit as most current vaccines are based on spike. Yet, most studies do not point in this direction. Finally, spike is also present in SARS-CoV-2 infection and nobody disputes neither the potential seriousness of COVID-19 nor the life-saving impact of vaccination.

### 8.3 Unconventional approaches

In natural infection, innate immunity can limit or abort the disease by creating a localized hyperinflammatory reaction driven by type-I IFNs which is followed by a resolutive T-cell response in most individuals with asymptomatic or mild COVID-19 presentations (224). Humoral neutralizing activity also helps to reduce viral spread but tends to be short-lived (229). The antibody response may be dispensable as demonstrated in patients with compromised humoral immunity who are capable of mounting an efficient antiviral T-cell reaction (29). This interpretation is also supported by finding antiviral T cell-mediated disease resolution without serum conversion in asymptomatic or mild COVID-19 (67). Instead, in severe cases, antibody production assumes an important fail-safe compensatory role that kicks in when hyperactivated T cells do not manage to clear the infection efficiently (26, 230, 231).

Vaccine-induced antibodies emulate the compensatory role of reducing virus spread observed in severe disease decreasing morbidity and lethality. However, viral clearance still relies on the concerted action with T cells (26). Indeed, to improve T-cell protection may be the way to go – considering that vaccines intended to produce neutralizing antibodies could not block infection by immune evasive VOCs like omicron but reduce severe disease (214, 220). This goal can be achieved by diversifying vaccine T-cell determinants as discussed previously and/or, alternatively, by altering the immune system “perception” of the pathogen’s identity with heterologous immunogens.

In the above context, one should consider harnessing the immune regulatory effect of trained immunity (232) and immune resetting (233) to compensate for pathogen immune evasion. Both strategies may offer cross-reactive protection. Trained immunity promotes the epigenetic and metabolic reprograming of innate immune cells and immune resetting relies on memory T-cell reactivation to promote heterologous protection. We have shown that the systemic and repeated recall of memory T-cell responses to unrelated antigens could revert the disease course in a model of polymicrobial high-grade sepsis (233). Microbial sepsis and COVID-19 do share many pathophysiological traits as we have discussed in detail elsewhere (234). It is worth noting that the secondary T-cell response is dominant over concurrent innate and primary adaptive heterologous immune reactions, thereby resetting the outcome to be more efficient and less inflammatory (62, 235–241).

We believe that immune resetting (adaptive) and trained immunity (innate) may lead to more effective anti-SARS-CoV-2 immune responses and may have a complementary role to vaccination, notably in case of emergence of highly evasive new variants by improving T-cell response. It is also relevant that immune resetting can rescue mice from sepsis-induced immunosuppression (233). This means that it has the potential to correct the selective but deleterious immune imprinting that certain SARS-CoV-2 variants may have on the response to subsequent infection by other VOCs. Ultimately, one could envisage that heterologous immune resetting would compensate for the reported T-cell subset singularities and turn the overall anti-SARS-CoV-2 response less hyperinflammatory and more resolutive. This hypothesis could be tested in a clinical trial in which a heterologous adaptive recall (and thereby an immune resetting) would be induced by the administration of a currently approved COVID-19 vaccine alongside a DTP booster for diphtheria, tetanus, and pertussis in previously immunized adults. The antigenic recall breadth could even be expanded (e.g., to cover hepatitis B). The latter vaccines have been well tested over the years and are associated to robust humoral responses but also induce a recallable T-cell memory (242–246).

In sum, we may be better off now than in early 2020. Bivalent mRNA vaccines, for instance, may curtail omicron subvariants present expansion (189, 190). Nevertheless, there will be newer challenges that require updating current vaccines to improve T-cell responses and attain better protection against COVID-19.

## 9 Conclusion

Just a few years back we knew close to nothing about a new virus that was bound to impose an enduring hit on humanity. The right mix of ingenuity, a bit of luck, and a lot of experience with related and unrelated pathogens, all converged into the development, testing, regulatory approval, and successful deployment of vaccines produced in multiple platforms. Two mRNA-, one adenoviral vector-, and two inactivated whole-virion-based vaccines were in the frontline to quench the infection waves unleashed by the ancestral SARS-CoV-2 strain and its first derivatives. And they did so brilliantly – with a success rate for protection from symptomatic infection that ranged roughly between 65-95%.

The assumed best correlate of protection was antibody neutralization. Indeed, there was evident neutralization activity against the ancestral virus in the sera collected from vaccinees. Nevertheless, it became apparent with the emergence of the first major drift variants – initially delta, then, omicron and its sublineages – that immune evasion was highly operative even in the context of a supposedly stable virus. A large array of mutations in the viral genome knocked one by one the neutralization epitopes, mostly on spike, favoring virus spread. Ultimately, vaccinees were no longer well-protected from infection but did not become proportionally more susceptible to severe disease and hospitalization. This observation led to the conclusion that a cellular antiviral immune response must have been preserved in the vaccinees who developed breakthrough infection, thereby blocking disease progression and promoting its resolution. Pre-clinical and clinical evidence indicated that T cells had a major role in the reported remaining protection.

There is no data from any large controlled clinical trial that compares prospectively the efficacy of the five most deployed vaccines, taking into account factors such as vaccinees’ age, dosing chronogram, and VOC subtyping. Thus, a head-to-head comparison is unfair. They all have performed well under the global health perspective so far. Moreover, these vaccines elicit a similarly powerful anti-viral CD4+ T cell response with polarization skewing towards the Th1 and cT_FH_ phenotypes. With the arguable exception of the inactivated whole-virion-based vaccines, the antiviral CD8^+^ T cell response elicited by the other major vaccines is equally evident with the generation of cells with cytotoxic potential and capable of producing IFN-γ as well as other effector cytokines. Moreover, in contrast to antiviral humoral neutralizing responses that wane quickly, T cell memory persists for 6-12 months post-vaccination and can be effectively reactivated.

At this stage of the pandemic, however, the extraordinary immune evasion capacity acquired by the latest omicron sublineages virtually turned all original vaccine preparations based on Wuhan-Hu-1 obsolete, and frankly unfit, to block virus spread in the community. Albeit protection from severe forms of the disease is retained in the general population, larger infection and reinfection rates are bound to overexpose the elderly and people with comorbidities to inauspicious outcomes. This grim picture motivated the WHO TAG-CO-VAC to advocate updating current vaccines to adjust them to the present viral sublineages. The development of bivalent vaccines represents the first substantive attempt to address this issue. Yet, despite the accelerated path for vaccine testing and approval presently implemented, newer more evasive viral sublineages appear constantly, and may take over the swarm by the time the latest bivalent products are fully deployed. Early CDC efficacy estimates for bivalent boosters offer some hope but we need more effort to be truly safe.

We believe that antigenic targets other than spike variants should be incorporated into the current vaccines and better formulations that increase cross-presentation and promote a designed T cell response should be considered. Moreover, the immune regulatory effect of trained immunity and immune resetting should be evaluated as part of a complementary strategy to vaccination to offer a new, cross-reactive, and improved T cell protection against immune evasive variants.

## Author contributions

AN and PC conceived and wrote all sections of the manuscript. MC contributed conceptually and for the writing of the section on vaccine production platforms. All authors contributed to the article and approved the submitted version.
